# Editorial: Integrative Structural Biology of Proteins and Macromolecular Assemblies: Bridging Experiments and Simulations

**DOI:** 10.3389/fmolb.2022.947582

**Published:** 2022-06-27

**Authors:** Paulo Ricardo Batista, Mario Oliveira Neto, David Perahia

**Affiliations:** ^1^ Programa de Computação Científica, Fundação Oswaldo Cruz, Rio de Janeiro, Brazil; ^2^ Departamento de Biofísica e Farmacologia, Instituto de Biociências de Botucatu, Universidade Estadual Paulista Júlio de Mesquita Filho, Botucatu, Brazil; ^3^ Laboratoire de Biologie et Pharmacologie Appliquée, École Normale Supérieure Paris-Saclay, Centre National de la Recherche Scientifique, Gif-sur-Yvette, France

**Keywords:** integrative modeling, enhanced sampling, hybrid simulations, protein structure, dynamics, CryoEM, SAXS, AFM

Despite continued advances in canonical experimental methods for protein structure determination, they all still possess significant limitations, which have become more evident when applied to challenge systems (Seffernick and Lindert, 2020). For example, this is the case with large transient macromolecular complexes, biomolecular machines, very flexible and intrinsically disordered proteins, or domains (Černý, 2017). Nevertheless, experimental structural information (from various sources and resolutions) can be joined through an integrative modeling approach, resulting in a more accurate model - or set of models. For this, experiments and predictions on how the subunits interact allow obtaining spatial restraints, which help determine the molecular architecture of proteins (Webb et al., 2018).

The success rate of resolving a protein structure is often inversely related to protein size and flexibility. Thus, predictions and measurements of protein dynamics are essential for effectively characterizing structural ensembles (Kuhlman and Bradley, 2019). Hence, hybrid simulations guided by experimental data efficiently sample an ensemble of function-relevant states (Seffernick and Lindert, 2020).

The goal of this Research Topic is to present applications of experimental and computational strategies regarding integrative structural approaches. This collection of nine articles explores experiments of high-speed atomic force microscopy (HS-AFM), small-angle X-ray scattering (SAXS), hydrogen-deuterium exchange mass spectrometry (HDX-MS), crosslinking MS (XL-MS), time-lapse microscopy, circular dichroism (CD), applied with complementary modeling tools, such as molecular docking, normal modes, molecular dynamics (MD), and hybrid enhanced sampling methods.

Soares et al. determined the molecular architecture of the complex formed between native DM64 glycoprotein and myotoxin II from *Bothrops asper* venom using an integrative structural modeling approach. Distance constraints from XL-MS guided the docking of DM64 domains to the myotoxin II crystal structure. SAXS and molecular dynamics simulations indicated that the complex is flexible and structured with an anisotropic shape. In addition, interprotein cross-links and limited hydrolysis analyses revealed inhibitory regions involved in the interaction with toxins.

Molza et al. have developed a complete integrative modeling pipeline that incorporates theoretical knowledge with advanced interactive and immersive modeling tools. As a result, it is possible to extract biologically relevant information by combining automated and human-driven interactive steps.

Proteins are polymers composed of simple units–amino acid residues. However, the dynamic folding process is highly complex (Scheraga et al., 2007). In the review published by Almeida et al. there is a theoretical discussion on the subject, presenting the relative contribution of the hydrophobic effect versus the stabilization of proteins by surface forces, which can sometimes go unnoticed.

Most proteins undergo conformational changes to perform their functions (Orellana, 2019). The article published by Pattanayak et al. monitored conformational changes of an outer membrane efflux protein resulting from the interaction with kanamycin. The bound structure was predicted by molecular docking, and the conformational changes occurred during the MD simulations. CD data suggests that the protein is less flexible and more compact in the presence of kanamycin.

The prediction/determination of relevant conformational states of proteins can be obtained by different methods. Dasgupta et al. modeled multiple conformations of a bacterial ClpB through HS-AFM images. The function of this protein relies on the interconversion between these different conformations. Therefore, even at low resolution, the knowledge of such states can help interpret dynamical-related properties. Still, on the subject, Fagnen et al. studied the dynamical properties of an engineered bacterial channel specifically designed to rest in an open conformation. Using an *in-silico* hybrid method that combines MD simulations and normal modes (Costa et al., 2015), global and local motions were captured and compared to HDX-MS experiments. The simulations also provided an estimation of the probability of the different opening states, agreeing with the electrophysiological experiments.

The major limitation of predicting protein dynamics using MD simulations is to sample rare long-time-scale events. Several enhanced sampling MD methods have been developed to address the problem (Yang et al., 2019). The article of Kaynak et al. presents a systematic comparison of the usefulness and limitations of four normal modes-based hybrid methods to explore the conformational space of proteins. They were applied to four well-studied proteins: triosephosphate isomerase, 3-phosphoglycerate kinase, HIV-1 protease, and HIV-1 reverse transcriptase.

Urazbaev et al. demonstrated that the microtubule growth in living cells could be approximated by a constant velocity with large stochastic fluctuations through time-lapse microscopy and mathematical modeling.

Ahmed et al. described an accurate prediction of residue burial aside from a quantitative prediction of specific residue contributions to protein stability and activity. However, this is a major challenge, especially in the absence of experimental structural information. Therefore, the authors resorted to the yeast surface display of a CcdB bacterial toxin saturation mutagenesis library to investigate the relationship between ligand binding and the level of expression of the displayed protein, with both *in vivo* solubility and *in vitro* thermal stability.
